# Effectiveness of treatment approaches for childhood exotropia: a systematic review

**DOI:** 10.7717/peerj.21416

**Published:** 2026-06-05

**Authors:** Saif Hassan Alrasheed, Saleh Alshammeri

**Affiliations:** Department of Optometry, College of Applied Medical Sciences, Qassim University, Buraydah, Saudi Arabia

**Keywords:** Exotropia, Child, Global health, Optical correction, Follow up, Symptom

## Abstract

**Background:**

Exotropia (XT), an outward ocular deviation, commonly affects children. Delayed treatment increases the risk of amblyopia and visual impairment. The aim of this systematic review is to evaluate the evidence on the effectiveness, indications, and clinical outcomes of different treatment modalities for childhood XT.

**Methods:**

A systematic search was performed in PubMed, Web of Science, ProQuest, Scopus, Google Scholar, EBSCO, and Medline in accordance with PRISMA 2020 guidelines. Articles published between 1997 and 2025 on treatment approaches for childhood XT were included. Treatment success was defined as a post-intervention deviation of ≤10 prism diopters (PD). The review protocol was registered in the International Prospective Register of Systematic Reviews (PROSPERO) under registration number CRD420251174532.

**Results:**

The final review included 34 studies from 11 countries, comprising 3,643 children with a mean age of 5.70 ± 2.24 years. The reviewed studies indicated that part-time occlusion and office-based vergence and anti-suppression therapy (OBVAT) achieved the highest short-term effectiveness rate of 86.8% after follow-up of five months. Overminus lenses therapy achieved a success rate of 80.63% with a follow-up of 2.15 years. Extraocular muscle surgery achieved a success rate of 70.81% with follow-up of 3.68 years, whereas botulinum toxin type A (BTX-A) injection showed a lower effectiveness rate of 66.86% after follow-up of 1.67 years.

**Conclusion:**

Multiple surgical and non-surgical options exist for managing childhood XT. Overminus lenses can improve distance control during testing, but deviation typically return to original state with non-overminus lenses, and their use requires monitoring for possible myopic progression. Surgery remains the primary treatment for constant XT, with bilateral lateral rectus recession and unilateral lateral rectus recession and medial rectus resection (R&R) showing similar short-term outcomes and long-term advantages for R&R. BTX-A offers a minimally invasive alternative for selected cases. Part-time occlusion and OBVAT improve short-term control, however durability and optimal protocols remain uncertain.

## Introduction

Strabismus is a common ocular disorder in early childhood, with an estimated prevalence ranging from 3% to 5% (Gore, Rath & Ganesh, 2023; Huang et al., 2025; Alrasheed et al., 2025). The condition has impact on facial aesthetics, can disrupt binocular vision function and lead to symptoms such as dizziness, nausea, and blurred vision, which may impair motor coordination including walking, balance and adversely affect the quality of life and developmental outcomes in children (Huang et al., 2025). Childhood exotropia (XT) is a form of strabismus characterized by a manifest outward deviation of one eye, and it affects approximately 0.1% to 3.7% of children (Ma & Scheiman, 2023). Delayed treatment of childhood XT is associated with a significantly increased risk of amblyopia, which may lead to persistent visual impairment during the developmental years (Wallace et al., 2018; Alrasheed & Aldakhil, 2024). Previous studies (Khorrami-Nejad, Akbari & Khosravi, 2018; Heydarian et al., 2020; Al-Rasheed, 2023) reported that approximately 48% to 92% of patients with XT present with intermittent exotropia (IXT). The prevalence of XT has been reported to be higher in countries located near the equator. It is notably more common in regions such as subequatorial Africa, the East Asia, and Middle East, where areas characterized by high levels of sunlight exposure compared to the prevalence of exodeviation in the United States and Central Europe (Heydarian et al., 2020; Jenkins, 1992).

Ansons & Davis (2008) described three clinical subtypes of XT divergence excess, convergence weakness, and basic XT based on the imbalance between convergence and divergence mechanisms (Ansons & Davis, 2008). The causes of childhood XT remain unclear; however, previous studies cited multiple contributing factors, including neuro-mechanical abnormalities, inadequate fusional mechanisms, a high accommodative convergence to accommodation (AC/A) ratio, refractive errors, and genetic predisposition (Mohney & Huffaker, 2003; Yetkin & Turkman, 2023; Zhou et al., 2025). Several hypotheses have been proposed regarding the development of XT. One widely held view suggests that a decompensated exophoria may progress to IXT, which can eventually become constant. Factors such as suppression, increased interpapillary distance, and reductions in accommodation and tonic convergence have been identified as potential contributors to the progression of XT (Wright & Strube, 2025). Previous studies (Nusz, Mohney & Diehl, 2006; Romanchuk, Dotchin & Zurevinsky, 2006) demonstrated that not all cases of IXT exhibit a progressive course; some remain stable over extended periods, while a smaller proportion of patients show improvement over time.

Management of childhood XT remains a complex and challenging aspect in clinical practice. Treatment is commonly initiated when binocular vision is affected or when the patient exhibits significant symptoms. The primary objective of intervention is to alleviate symptoms and reduce the frequency of manifest deviation, either by decreasing the angle of the strabismus or by enhancing the fusional vergence. Various modalities exist for the management of childhood XT, including both surgical and non-surgical approaches. Non-surgical interventions include overminus lens therapy, part-time occlusion therapy, prism correction, and office-based vergence and anti-suppression therapy (OBVAT), each aiming to improve ocular alignment and enhance binocular visual function. The rationale for this systematic review is to integrate research findings on childhood XT management and to evaluate the effectiveness and indications of available treatment strategies. Thus, the aim of this systematic review is to evaluate the evidence on the effectiveness, indications, and clinical outcomes of different treatment modalities for childhood XT.

## Methods

### Study design

This systematic review was conducted in accordance with the procedures outlined in the Preferred Reporting Items for Systematic Reviews (PRISMA 2020) (Page et al., 2021). The review protocol was registered in the International Prospective Register of Systematic Reviews (PROSPERO) under registration number CRD420251174532. The researchers were performing a comprehensive literature search across various electronic databases, including the Web of Science, Scopus, PubMed, Google Scholar, ProQuest, Medline, and EBSCO. The search included original studies published between January 1997 and November 2025. The present study used timeframe 1997–2025 to capture all relevant published studies on childhood XT and to reflect changes in treatment approaches over time. The methodological quality of each study included in this review will evaluate using the assessment tool developed by Downs & Black (1998). In addition, each selected study goes through a rigorous appraisal process and will assign a quality score ranging from 1 to 10, as shown in Table 1. The present review encompassed a broad spectrum of original studies to assess various therapeutic interventions for different subtypes of childhood XT. The included studies were drawn from multiple countries and involved pediatric populations across a range of age groups.

**Table 1:** Characteristics of studies assessed childhood exotropia management.

**Author and published** **year**	**Country**	**Age group** **(years)**	**Age** **(Mean (SD)**	**Study design**	**Sample** **size**	**Quality assessment** **score**
Abdelfatah et al. (2018)	Egypt	2–10	4.7 ± 2.6	RCT	60	10
Akbari, Mehrpour & Mirmohammadsadeghi (2021)	Iran	3–8	5.0 ± 1.3	RCT	76	10
Pediatric Eye Disease Investigator Group et al. (2015)	USA	3–12	6.1 ± 1.7	RCT	201	10
Pediatric Eye Disease Investigator Group et al. (2014)	USA	3–10	6.0 ± 2.0	RCT	358	10
Ale Magar et al. (2022)	Australia	5–15	6.8 ± 2.5	RCT	141	10
Pediatric Eye Disease Investigator Group et al. (2016)	USA	3–6	5.1 ± 1.1	RCT	58	10
Chen et al. (2021)	USA	3–10	6.3 ± 2.1	RCT	386	10
Ma et al. (2024)	China	6–17	–	RCT	40	9
Feng et al. (2021)	China	3-6	4.9 ± 1.0	RCT	60	10
Paula et al. (2009)	Brazil	<11	4.20 ± 2.8	Retrospective	21	9
Bayramlar et al. (2017)	Turkey	3–14	6.8 ± 3.3	Retrospective	19	9
Issaho, Wang & Weakley Jr (2017)	Brazil	–	6.8 ± 2.6	Retrospective	136	9
Repka et al. (2020a)	USA	3–10	6.2 ± 2.0	RCT	197	10
Pediatric Eye Disease Investigator Group et al. (2019)	USA	3–10	6.2 ± 2.0	RCT	197	10
Wang et al. (2013)	China	3–15	–	Retrospective	85	9
Spierer & Spierer (2021)	Israel	3.5–11	6.4 ± 1.9	Retrospective	58	9
Bang, Cho & Lee (2017)	Korea	–	7.22 ± 4.84	Retrospective	99	9
Donahue et al. (2024)	USA	3–10	–	RCT	123	10
Kushner (1998)	USA	5–15	–	RCT	36	9
Yousef et al. (2025)	Egypt	3–10	–	Randomized comparative	40	9
Elkhawaga, Kassem & Elkamshoushy (2023)	Egypt	6–12	–	Prospective	36	9
Huang & Li (2022)	China	1–7	3.92 ± 2.02	Retrospective	25	9
Jiang et al. (2023)	China	12–17	–	Retrospective	19	9
Spencer et al. (1997)	USA	3 to 144 months	–	Case-controlled	32	9
Li & Wu (2008)	China	4–12	–	Prospective	60	9
Tugcu et al. (2025)	Turkey	5–17	–	Prospective	34	9
Su et al. (2022a)	China	2–17	7.1 ± 2.5	Prospective	72	10
Kunduracı et al. (2022)	Turkey	5–17	–	Prospective	74	9
Lino, De Aguiar & Cunha (2025)	Portugal	5–17	11.19 ± 3.73	Retrospective	258	10
Hatt et al. (2023)	USA	3–10	–	RCT	306	10
Ma et al. (2019)	China	6–17	–	Prospective	14	9
Alizadeh et al. (2023)	Iran	<7	2.25 ± 0.74	Prospective	106	9
Mangad et al. (2018)	India	1–5 years	3.6 ± 1.633	Prospective	53	9
Abri Aghdam et al. (2021)	Iran	<6	3 ± 2.6	Retrospective	163	9
All			5.70 ± 2.24		3,643	9.41

**Notes.**

RCT Randomized controlled trial

### Search policy and selection criteria

The search strategy for this systematic review was employed by Boolean operators (AND/OR) to combine relevant keywords effectively. A comprehensive literature search was conducted across six electronic databases to identify relevant studies published between January 1997 and November 2025. The search strategy was utilized Medical Subject Headings (MeSH) and relevant keywords, combined using Boolean operators (AND/OR), including the following terms: (Refractive correction OR Optical correction OR Orthoptic exercises OR Anti-suppression therapy OR overminus therapy OR over correction) OR (Surgical interventions OR Extraocular muscle surgery) OR (Botulinum toxin therapy OR Drug therapy) OR (Prism therapy OR Optical correction) AND Childhood XT (Primary XT OR IXT OR Constant XT OR Near XT OR Distance XT OR Early-onset XT).

### Eligibility and inclusion criteria

The scope of this review was limited to scholarly articles published in peer-reviewed journals and written in English. Studies were included if they focused on the management of childhood XT, specifically addressing various treatment modalities for different subtypes of the condition. Articles were excluded if they did not focus on pediatric populations or did not evaluate therapeutic interventions for managing of childhood XT. Additionally, non-peer-reviewed publications such as conference abstracts, editorial commentaries, perspective, meeting proceedings, pilot studies, and articles lacking essential data collection were excluded from this systematic review.

### Data extraction process

The titles and abstracts of all identified articles were independently screened by the authors using a standardized data extraction form. This form was used to systematically collect relevant information, including the first author’s name, year of publication, country in which the study was conducted, participant characteristics (such as age and sample size), the specific type of childhood XT addressed, the treatment modalities employed, and the reported success rates of each intervention. In the present review, treatment success was defined as a post-intervention of ocular deviation of equal or less than 10 prism diopters (PD), following management with optical correction, orthoptic exercises, occlusion therapy, extraocular muscle surgery, or botulinum toxin type A (BTX-A) injection. To ensure consistency and transparency throughout the review process, predefined standards and protocols were established to guide the selection and evaluation of studies. In instances where discrepancies arise among reviewers, structured procedures were followed to facilitate open communication and the resolution of disagreements. These protocols were designed to promote objective decision-making and consensus-building, thereby enhancing the reliability and rigor of the systematic review. Any discrepancies between reviewers regarding study selection, data extraction, or quality assessment were addressed through discussion and resolved based on predefined, objective criteria rather than personal assumptions or subjective opinions. When consensus could not be reached, an independent eye-care expert was consulted to provide judgment and facilitate resolution.

### Risk of bias assessment

The methodological quality of each study included in this systematic review was assessed using the Downs and Black checklist (Downs & Black, 1998), a validated tool for evaluating both randomized and non-randomized studies, as shown in Table 1. Two independent reviewers will conduct the risk of bias assessment for each included study. Any discrepancies or disagreements arising during the evaluation process were resolved through discussion. If consensus cannot be reached, an independent eye-care expert was consulted to provide an independent judgment and facilitate resolution.

## Results

### Study features

**Figure 1:**
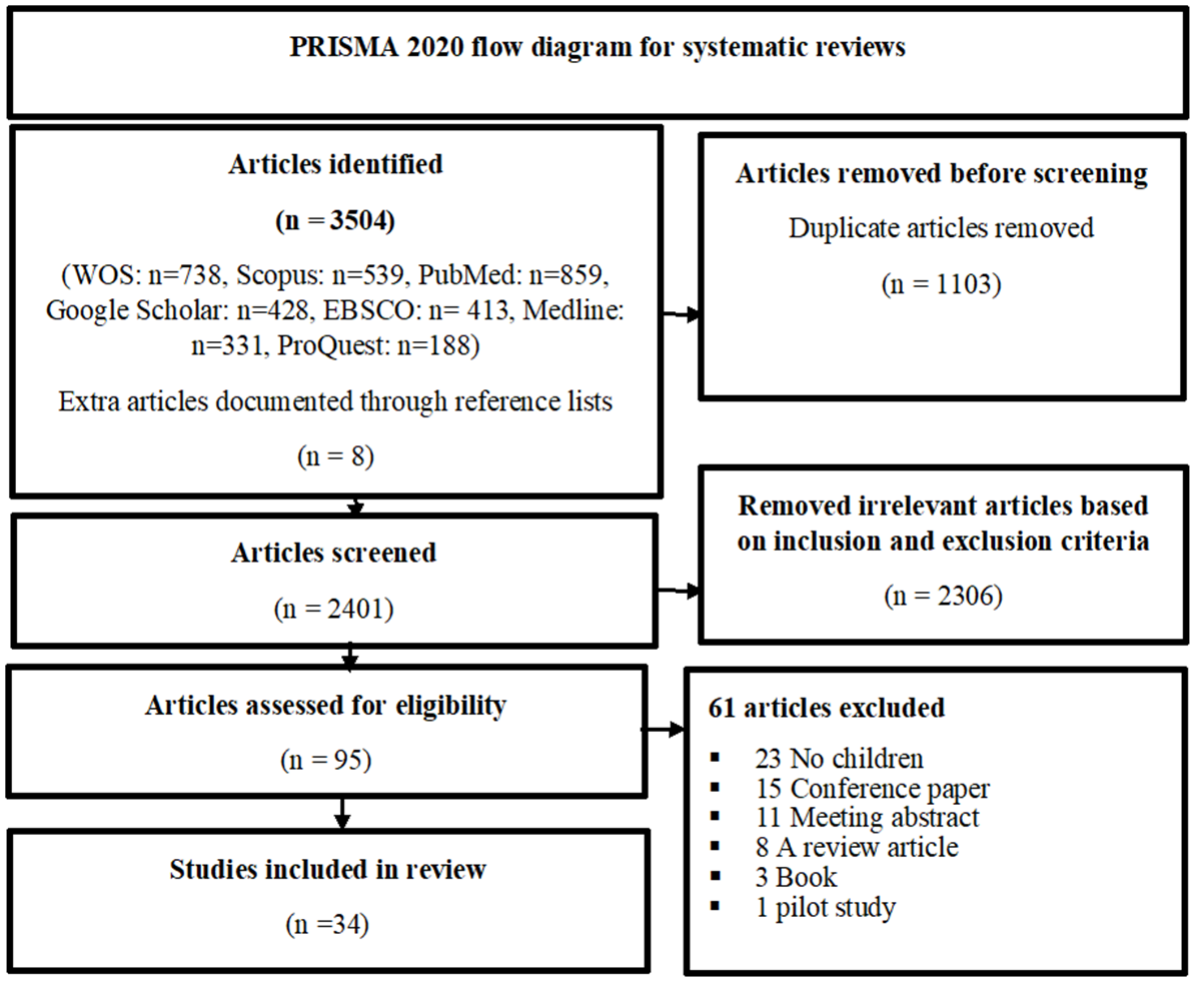
PRISMA 2020 flow diagram for systematic review.

A total of 3,504 studies were initially identified as shown in Fig. 1. After removing duplicates, 2,401 titles were screened, of which 2,306 were excluded following abstract review for not meeting the inclusion criteria. Furthermore, 61 studies were excluded because they did not include children, were conference abstracts, review articles, or book chapters, or lacked sufficient extractable information or pilot study, as presented in Fig. 1. Thus, this systematic review comprised 34 quality-assessed studies (Abdelfatah et al., 2018; Akbari, Mehrpour & Mirmohammadsadeghi, 2021; Pediatric Eye Disease Investigator Group et al., 2015; Pediatric Eye Disease Investigator Group et al., 2014; Ale Magar et al., 2022; Pediatric Eye Disease Investigator Group et al., 2016; Chen et al., 2021; Ma et al., 2024; Feng et al., 2021; Paula et al., 2009; Bayramlar et al., 2017; Issaho, Wang & Weakley Jr, 2017; Repka et al., 2020a; Pediatric Eye Disease Investigator Group et al., 2019; Wang et al., 2013; Spierer & Spierer, 2021; Bang, Cho & Lee, 2017; Donahue et al., 2024; Kushner, 1998; Yousef et al., 2025; Elkhawaga, Kassem & Elkamshoushy, 2023; Huang & Li, 2022; Jiang et al., 2023; Spencer et al., 1997; Li & Wu, 2008; Tugcu et al., 2025; Su et al., 2022a; Kunduracıet al., 2022; Lino, De Aguiar & Cunha, 2025; Hatt et al., 2023; Ma et al., 2019; Alizadeh et al., 2023; Mangad et al., 2018; Abri Aghdam et al., 2021) conducted across 11 countries, as summarized in Table 1. The included studies, published between 1997 and 2025, represented a combined sample of 3,643 children with a mean age of 5.70 ± 2.24 years, as shown in Table 1. Most studies defined treatment success as a postoperative or post-intervention ocular deviation of equal or less than 10 PD.

The studies reviewed (Abdelfatah et al., 2018; Ale Magar et al., 2022; Pediatric Eye Disease Investigator Group et al., 2016; Chen et al., 2021; Ma et al., 2024; Feng et al., 2021; Paula et al., 2009; Bayramlar et al., 2017; Huang & Li, 2022; Jiang et al., 2023; Spencer et al., 1997; Li & Wu, 2008), summarized in Table 2, indicated that overminus correction achieved a success rate of 80.63% in managing childhood XT, with an average follow-up of 2.15 years. Similarly, the studies reviewed (Issaho, Wang & Weakley Jr, 2017; Repka et al., 2020a; Pediatric Eye Disease Investigator Group et al., 2019; Wang et al., 2013; Spierer & Spierer, 2021; Bang, Cho & Lee, 2017; Donahue et al., 2024; Kushner, 1998; Yousef et al., 2025; Elkhawaga, Kassem & Elkamshoushy, 2023; Huang & Li, 2022; Lino, De Aguiar & Cunha, 2025), presented in Table 3, showed that extraocular muscle surgeries, including bilateral lateral rectus recession (BLRs), unilateral lateral rectus recession with medial rectus medial rectus resection(R&R), unilateral lateral rectus recession, bilateral lateral rectus fenestration, and augmented lateral rectus recession, achieved overall success rate of 70.81% with an average follow-up of 3.68 years. In contrast, the studies reviewed (Jiang et al., 2023; Spencer et al., 1997; Li & Wu, 2008; Tugcu et al., 2025; Su et al., 2022a; Kunduracıet al., 2022), summarized in Table 4, reported that BTX-A injection demonstrated a lower effectiveness rate of 66.86% after an average follow-up of 1.67 years. The studies reviewed (Abdelfatah et al., 2018; Akbari, Mehrpour & Mirmohammadsadeghi, 2021; Pediatric Eye Disease Investigator Group et al., 2015; Pediatric Eye Disease Investigator Group et al., 2014; Ma et al., 2024; Hatt et al., 2023; Ma et al., 2019), summarized in Table 5, indicated that occlusion and OBVAT achieved the highest effectiveness rate of 86.8% after an average follow-up of 0.42 years (about 4 months). Finally, comparative summary of treatment of the childhood XT modalities including follow-up duration, outcome measures, and mean effectiveness rates presented in Table 6.

**Table 2:** Studies used optical correction for management of childhood exotropia.

**Author and published** **year**	**Types of esotropia**	**Treatment** **method**	**Follow** **up(year)**	**Outcome measure**	**Effective** **rate**	**Treatment outcome and conclusion**
Abdelfatah et al. (2018)	IXT	Overminus	0.33	<10 PD	90%	Overminus therapy has revealed greater efficacy in controlling IXT compared to occlusion therapy.
Ale Magar et al. (2022)	IXT	Overminus	1.25	Stereopsis	100%	Overminus therapy was found to be effective in enhancing distance control and improving stereopsis.
Pediatric Eye Disease Investigator Group et al. (2016)	IXT	Overminus	0.17	<10 PD	59%	Overminus therapy improved distance control.
Chen et al. (2021)	IXT	Overminus	1.00	Stereopsis	88.4%	Overminus therapy improved distance exotropia control but led to a greater myopic shift, with the effect not sustained after treatment stop.
Feng et al. (2021)	IXT	Overminus	1.00	Control and Stereopsis	71.4%	Overminus spectacles with prism significantly improved IXT control and stereopsis, without inducing myopia
Paula et al. (2009)	IXT	Overminus	7.00	Refractive change	–	Overcorrecting minus lens therapy for IXT did not induce refractive changes, regardless of age, treatment duration, initial spherical equivalent, or degree of overcorrection.
Bayramlar et al. (2017)	XT	Overminus	1.50	<10 PD	84%	Overcorrecting minus lens therapy demonstrates a reasonable medium-term success rate and may be considered a primary treatment option for intermittent XT.
Alizadeh et al. (2023)	IXI	Overminus	5.00	<10 PD	79.24%	The management of IXT through the use of overminus lenses represents a safe and effective method for reducing the risk of progression to constant XT.
Mangad et al. (2018)	IXI	Overminus	1.00	<10 PD	86.8%	Overminus therapy significantly improves IXT control in children under 5 and is recommended as an initial treatment.
Abri Aghdam et al. (2021)	IXI	Overminus	3.20	<10 PD	66.8%	Overminus lens therapy effectively improves IXT control in young children and may delay the need for surgery.
All			2.15		80.63%	

**Notes.**

IXT intermittent exotropia XT exotropia

**Table 3:** Studies used surgical management for childhood exotropia.

**Author and published** **year**	**Types of esotropia**	**Treatment** **method**	**Follow** **up (year)**	**Outcome measure**	**Effective** **rate**	**Treatment outcome and conclusion**
Issaho, Wang & Weakley Jr (2017)	IXT	Bilateral LR recession	4	<10 PD	64.5%	Surgical intervention for IXT can be safely performed in children under four years of age and may yield superior motor outcomes compared to older patients.
Repka et al. (2020a)	IXT	Bilateral LR recession	3	<10 PD	87%	Children aged 3 to <5 years had better surgical outcomes for IXT than those aged 5 to <11 years, with no other predictive factors identified.
Pediatric Eye Disease Investigator Group et al. (2019)	IXT	Bilateral LR recession VS unilateral LR recession- MR resection	3	<10 PD	87%	No significant difference in suboptimal outcomes at three years was found between Bilateral LR recession and unilateral LR recession- MR resection.
Wang et al. (2013)	IXT	Bilateral LR recession	0.5	<10 PD	65.8%	–
Wang et al. (2013)	IXT	Unilateral LR recession- MR resection	0.5	<10 PD	85.1%	Unilateral LR recession with MR resection is more effective long-term than bilateral LR recession for basic-type IXT in children
Spierer & Spierer (2021)	IXT	Unilateral LR recession	12	≤10 PD	86.2%	Unilateral LR recession is effective for moderate-angle exotropia in children.
Bang, Cho & Lee (2017)	IXT	Bilateral LR recession	5	≤10 PD	54.1%	Five-year surgical outcomes for IXT were comparable between BLR and RR, with no significant differences in success or reoperation rates.
Bang, Cho & Lee (2017)	IXT	Unilateral LR recession- MR resection	5	≤10 PD	41.9%	Five-year surgical outcomes for IXT were comparable between Bilateral LR recession and Unilateral LR recession- MR resection, with no significant differences in success or reoperation rates.
Donahue et al. (2024)	IXT	Bilateral LR recession	8	≤10 PD	68%	No significant different in outcome between Bilateral LR recession and Unilateral LR recession- MR resection
Donahue et al. (2024)	IXT	Unilateral LR recession- MR resection	8	≤10 PD	53%	–
Kushner (1998)	IXT	Bilateral LR recession	1	≤10 PD	52%	–
Kushner (1998)	IXT	Unilateral LR recession- MR resection	1	≤10 PD	82%	Evidence suggests that recess–resect procedures are recommended for managing basic-type IXT.
Yousef et al. (2025)	IXT	Bilateral LR recession	3	≤10 PD	70%	–
Yousef et al. (2025)	IXT	Fenestration LR	3	≤10 PD	50%	Bilateral LR recession had better outcome than Fenestration LR
Elkhawaga, Kassem & Elkamshoushy (2023)	IXT	Bilateral fenestration LR	0.5	≤8PD	81%	Bilateral LR fenestration achieved successful outcomes in most patients, significantly reducing deviation at both distance and near fixation.”
Huang & Li (2022)	PIE	Augmented LR recession	1	≤10 PD	81.0%	Augmented LR recession is an effective and safe procedure for treating large-angle PIE.
Lino, De Aguiar & Cunha (2025)	IXT	Bilateral LR recession	4	≤10 PD	92.2%	Preoperative deviation angle was the strongest predictor, with smaller angles associated with better surgical outcomes.
All			3.68		70.81%	

**Notes.**

LR Lateral rectus PD Prism diopter MR Medial rectus PIE primary infantile exotropia

**Table 4:** Studies used Botulinum Toxin for management of childhood exotropia.

**Author and published** **year**	**Types of esotropia**	**Treatment** **method**	**Follow** **up (year)**	**Outcome measure**	**Effective** **rate**	**Treatment outcome and conclusion**
Jiang et al. (2023)	XT	Bilateral LR BT injection	1.00	<10 PD and stereopsis	71.4%	BTX-A appears to be an effective option for managing exotropia
Spencer et al. (1997)	IXT	Bilateral LR BT injection	4.00	<10 PD	69%	BTX-A is effective in treatment of IXT particularly in patients aged 2–4.5 years.
Li & Wu (2008)	IXT	Bilateral LR BT injection	0.50	<10 PD	76.67%	BTX-A injection is a rapid, minimally invasive, and effective treatment for IXT
Tugcu et al. (2025)	Consecutive XT	Bilateral LR BT injection	2.00	<10 PD	73.5%	BTX-A injection can be an effective and harmless management option in consecutive XT.
Su et al. (2022a)	IXT	Bilateral LR BT injection	0.50	<10 PD	52.8%	BTX-A is as effective as surgery for treating IXT in children, but fusion recovery is lower.
Kunduracı et al. (2022)	IXT	Bilateral LR BT injection	2.00	<10 PD	56.7%	BTX-A is an effective, less invasive, and time-efficient alternative to surgery for small to medium angle IXI
All			1.67		66.68%	

**Notes.**

BTX-A Botulinum toxin type A

**Table 5:** Studies used occlusion and vision therapy for management of childhood exotropia.

**Author and Published** **year**	**Types of esotropia**	**Treatment** **method**	**Follow** **up(year)**	**Outcome measure**	**Effective** **rate**	**Treatment outcome and conclusion**
Abdelfatah et al. (2018)	IXT	Occlusion	0.33	<10 PD	74	Alternate patching 4 h daily is effective for IXT, but poor compliance reduces its success.
Akbari, Mehrpour & Mirmohammadsadeghi (2021)	IXT	Occlusion	0.5	Stereoacuity	100	Alternate patching for 2 h daily, with dominant eye 5 days and the non-dominant eye 2 days weekly, appears effective in improving control of childhood IXT.
Pediatric Eye Disease Investigator Group et al. (2015)	IXT or XT	Occlusion	0.5	<10 PD	97.8	Part-time patching for 3 h daily over a period of 5 months as an effective treatment for IXT in children.
Pediatric Eye Disease Investigator Group et al. (2014)	IXT	Occlusion	0.5	<10 PD	99	Part-time patching for 3 h daily over a period of 5 months as an effective treatment for IXT in children.
Ma et al. (2024)	IXT	OBVAT	0.35	<10 PD	75	Office-based vergence and anti-suppression therapy demonstrated promising results in controlling distance IXT.
Hatt et al. (2023)	IXT	Occlusion	0.5	<10 PD	75	Part-time patching for 3 h daily may enhance distance control in children with IXT.
Ma et al. (2019)	IXT	OBVAT	0.25	–	–	Twelve weeks of office-based vergence/accommodative therapy with home reinforcement significantly improved distance control and reduced near IXT.
All			0.42		86.8%	

**Notes.**

IXT intermittent exotropia OBVAT Office-based vergence and anti-suppression therapy

**Table 6:** Comparative summary of treatment modalities including follow-up duration, outcome measures, and mean effectiveness rates.

**Treatment modality**	**No.**	**Follow-up range (years)**	**Primary outcome measures**	**Effective rate**	**Treatment outcome & conclusion**
Overminus Lenses	10	0.17–7.0	≤10 PD deviation control and stereopsis	80.63%	Overminus lenses can improve distance control during testing, but deviation typically return to original state with non-overminus lenses, and their use requires monitoring for possible myopic progression.
Surgical Management	17	0.5–12	≤10 PD deviation control and stereopsis	70.81%	Children under five years demonstrate better motor and surgical outcomes in the management of IXT. Children with smaller preoperative deviation angles predicting higher success rates. Extraocular muscle surgery continues to be the primary treatment for constant XT mainly primary infantile XT, with both BLRc and R&R showing similar short-term outcomes, whereas long-term evidence suggests potential advantages for R&R.
Botulinum Toxin (BTX-A)	6	0.5–4.0	≤10 PD deviation control, stereopsis	66.68%	BTX-A provides a minimally invasive option for selected cases, offering variable control and satisfactory motor alignment. However, its sensory outcomes are less predictable compared with standard surgical approaches, and long-term stability generally remains inferior to extraocular muscle surgery.
Occlusion and BVAT	7	0.33–0.5	≤10 PD deviation, stereopsis	86.8%	Short-term evidence indicates that part-time occlusion and OBVAT can improve control and binocular function in IXT; however, uncertainties remain regarding the durability of the management effects, optimal treatment duration, and appropriate patient selection.

**Notes.**

IXT intermittent exotropia OBVAT Office-based vergence and anti-suppression therapy

## Discussion

### Optical correction for management of childhood XT

#### Overminus lens therapy

The present systematic review shows that optical interventions particularly overminus lens therapy remain the most effective nonsurgical method for enhancing distance control in children with IXT. Across studies (Abdelfatah et al., 2018; Ale Magar et al., 2022; Pediatric Eye Disease Investigator Group et al., 2016; Chen et al., 2021; Ma et al., 2024; Feng et al., 2021; Paula et al., 2009; Bayramlar et al., 2017; Huang & Li, 2022; Jiang et al., 2023; Spencer et al., 1997; Li & Wu, 2008), success rates ranged from 59% to 100%, with a pooled effectiveness of about 80.63%, as presented in Tables 2 and 6. These findings are consistent with the PEDIG RCT, which demonstrated significant improvement in distance control at 12 months using −2.50 D overminus lenses (Chen et al., 2021). However, the durability and refractive safety of overminus therapy require careful consideration, particularly in young children. The PEDIG trial found that treatment effects diminished after tapering and discontinuation, and that children receiving overminus lenses experienced a greater myopic shift (Chen et al., 2021). Furthermore, three-year extension study confirmed that the myopic shift persisted but did not continue to increase after treatment cessation, suggesting that refractive impact is concentrated within the first year (Chen et al., 2024). Accordingly, refractive monitoring and clear counseling regarding potential myopic progression are recommended. Earlier retrospective work by Paula et al. (2009) reported no refractive changes with overminus therapy, but discrepancies with PEDIG RCT likely reflect smaller samples and less standardized refraction methods. More recent RCT study incorporating customized algorithms for determining overminus power reported high tolerability and limited refractive impact, supporting individualized treatment approaches (Ale Magar et al., 2022). A recent meta-analysis further supports the motor benefits of overminus therapy, showing improvement in control and reduction in exodeviation magnitude, although sensory outcomes such as near stereopsis show limited or inconsistent improvement (Song et al., 2023a). Overall, overminus lenses remain a valuable nonsurgical option for managing IXT; however, their use should be guided by careful consideration of the balance between short-term motor benefits and potential refractive risks, with treatment individualized to each child’s clinical profile.

#### Base in prism therapy

In this systematic review, prism therapy appears less effective as a primary treatment for childhood XT. A pilot RCT found no improvement in distance control with base-in prism compared to refractive correction alone (Summers et al., 2023). In contrast, combination therapy using overminus lenses with small base-in prism confirmed improved deviation control and stereopsis without inducing myopia over 12 months follow up (Feng et al., 2021). Overall, current evidence supports overminus lens therapy as an effective short-term intervention for childhood IXT, particularly for delaying surgical management. Clinicians should provide counseling regarding refractive risks, implement regular monitoring for myopic progression, and consider individualized minus power or combined optical strategies to optimize the balance between efficacy and safety. Future research should prioritize the development of customized management protocols, evaluation of combination approaches, and assessment of long-term effects on refractive and sensory outcomes.

#### Extraocular muscle surgery management for childhood XT

The studies (Issaho, Wang & Weakley Jr, 2017; Repka et al., 2020a; Pediatric Eye Disease Investigator Group et al., 2019; Wang et al., 2013; Spierer & Spierer, 2021; Bang, Cho & Lee, 2017; Donahue et al., 2024; Kushner, 1998; Yousef et al., 2025; Elkhawaga, Kassem & Elkamshoushy, 2023; Huang & Li, 2022; Lino, De Aguiar & Cunha, 2025) summarized in Tables 3 and 6 collectively confirm that two-muscle surgery remains the primary management option for childhood XT, with bilateral lateral rectus recession (BLRc) and unilateral lateral rectus recession and medial rectus resection (R&R) being the most used techniques. Nevertheless, there is no clear consensus regarding the relative efficacy of these two surgical techniques in terms of postoperative alignment, residual or recurrent of XT, and the risk of consecutive esotropia. The variability in reported outcomes may reflect differences in study design, surgical technique, and an incomplete understanding of the natural progression of the disorder (Chougule & Kekunnaya, 2019). The reported success rates for extraocular muscle surgery in childhood XT, commonly defined as achieving postoperative alignment within 10 D, showed wide variability ranging from 41.9% to 92%. This variation reflects significant heterogeneity across studies, such as differences in surgical techniques (BLRc, R&R, BLR fenestration), patient characteristics, baseline control, and type of XT. Additionally, outcomes are influenced by inconsistent follow-up durations short-term studies (Wang et al., 2013; Kushner, 1998; Elkhawaga, Kassem & Elkamshoushy, 2023; Huang & Li, 2022) often report higher success along with variations in outcome definitions and measurement methods. Together, these factors limit the comparability of results across cohorts and highlight the need for cautious interpretation of reported success rates. Recent study (Repka et al., 2020b) indicates that, among children with IXT, younger age at surgery (3 to <5 years) is associated with more favorable outcomes. The PEDIG RCT study in children with basic-type IXT showed no statistically significant difference between BLRc and R&R at three years when assessed using suboptimal outcome measure (including recurrent XT, consecutive esotropia, stereoacuity loss, or need for reoperation), thus supporting the short-term equivalence of these procedures (Pediatric Eye Disease Investigator Group et al., 2019). Furthermore, five-year surgical outcomes for IXT were comparable between BLRc and R&R, with no significant differences in success or reoperation rates (Bang, Cho & Lee, 2017). In contrast, eight-year follow-up of the RCT revealed notable secondary differences: The R&R procedure was associated with fewer reoperations and a higher proportion of complete resolution compared with BLRc, despite the primary composite endpoint not reaching statistical significance. These findings suggest potential long-term advantages of R&R at the surgical doses utilized in the study (Donahue et al., 2024). Interestingly, a recent study (Oke et al., 2024) revealed that children undergoing R&R were less likely to require reoperation within five years compared with those treated with BLRc. These findings underscore the need for further research to identify modifiable risk factors for reoperation, with the goal of reducing surgical burden and improving long-term outcomes in children with IXT. Building on these findings, a long-term retrospective cohort study with a mean follow-up of approximately 10 years also favored R&R over BLRc, demonstrating greater sustained success and lower recurrence rates when follow-up extended beyond the early postoperative period (Kim, Yang & Hwang, 2021). Furthermore, previous meta-analysis evidence has similarly suggested higher success rate and lower recurrence with R&R in IXT, while acknowledging heterogeneity and study design limitations (Sun, Zhang & Chen, 2018).

In a secondary analysis of PEDIG RCT data, younger age (3 to <5 years) was associated with a reduced risk of suboptimal outcome at three years. In contrast, other baseline characteristics including magnitude of deviation, control score, fixation preference, and near stereoacuity did not demonstrate independent predictive value within this dataset (Repka et al., 2020b). More recent study highlights that the preoperative deviation angle was the strongest predictor, with smaller angle associated with better surgical outcomes (Lino, De Aguiar & Cunha, 2025). Moreover, recent findings underscore the predictive significance of early postoperative ocular alignment. Evidence suggests that achieving a mild degree of overcorrection during the first postoperative week is associated with an increased likelihood of long-term surgical success (Srimanan & Keokajee, 2025). In cases of primary infantile XT with large deviation angles, the use of augmented BLRc using a hemi hang-back technique has demonstrated high rates of motor success without inducing abduction deficits in small case series. These findings suggest that this method may represent a practical alternative in situations where standard surgical give risk of under-correction (Huang & Li, 2022). Fenestration of the lateral rectus muscle has recently been proposed as an alternative to conventional recession techniques. In a prospective pediatric cohort, BLR fenestration for IXT achieved approximately 81% success at six months, with significant improvements in both distance and near deviations and no major complications reported (Elkhawaga, Kassem & Elkamshoushy, 2023). These findings indicate that BLR fenestration may offer a safe and effective option in selected cases, although further studies with larger sample sizes and longer follow-up are warranted to confirm its long-term efficacy and safety.

#### Botulinum toxin type A in management of childhood XT

This study synthesizes outcomes of BTX-A injected into the lateral rectus (LR) muscles for the management of childhood XT, primarily IXT, with one series addressing consecutive XT (Tugcu et al., 2025). Across six cohorts summarized in Tables 4 and 6 (Jiang et al., 2023; Spencer et al., 1997; Li & Wu, 2008; Tugcu et al., 2025; Su et al., 2022a; Kunduracıet al., 2022), the pooled effective rate was approximately 66.7%, based on a motor success criterion of ≤10 PD only study assessed stereoacuity. These findings are consistent with both historical and contemporary evidence, indicating that BTX-A injection can achieve short to medium-term ocular alignment in pediatric patients with small to moderate-angle XT (Jiang et al., 2023; Spencer et al., 1997; Li & Wu, 2008; Tugcu et al., 2025; Su et al., 2022a; Kunduracıet al., 2022).

Spencer et al. (1997) reported 69% success rate following bilateral LR BTX-A injections and highlighted greater responsiveness in children aged 2–4.5 years, an age range that may facilitate- motor adaptation and sensory recovery. This age-related advantage and overall success rate are consistent with the findings of our synthesis. In contrast, Li & Wu (2008) conducted a prospective comparison of BTX-A injection and surgical intervention at six months and reported no statistically significant difference in success rates (76.7% *vs* 90.0%). These findings suggest that BTX-A injection can be a minimally invasive alternative for selected IXT cases, though its effectiveness may be slightly lower than surgery. Su et al. (2022a) reported modern comparative data in children with IXT, showing six months success rate of 52.8% for BTX-A injection *versus* 66.7% for surgery (*P* = 0.13), indicating- similar motor outcomes but significantly poorer fusion recovery with BTX-A (68.1% *vs* 95.8%, *P* < 0.001). This sensory difference highlights that, although BTX-A commonly achieves alignment in small to moderate deviations, restoring stable binocularity is less predictable compared to surgery as an important consideration when counseling families.

The American academy of ophthalmology reports that extraocular muscle injection of BTX-A can achieve motor alignment rates comparable to those obtained with extraocular muscle surgery in cases of nonparalytic, nonrestrictive horizontal strabismus. However, sensory outcomes following BTX-A injection are considerably more variable than extraocular muscles surgery. Whereas some children regain functional binocularity after treatment, others show limited or no improvement in sensory outcomes, and the likelihood of achieving stable sensory fusion appears less predictable than with surgery. In contrast, surgical correction has a more established track record of producing consistent sensory recovery, particularly in young children, where early restoration of binocularity is critical. Additionally, the need for multiple BTX-A injection further contributes to outcome variability, underscoring the uncertainty surrounding its sensory benefits compared to the more documented sensory outcomes of extraocular muscle surgery.

Binenbaum et al. (2021) and Kunduracıet al. (2022) reported a motor success rate of 56.7% in children with IXT treated using bilateral LR BTX-A injections. Their findings suggest that BTX-A injection represents an effective, less invasive, and time-efficient alternative to conventional surgery for managing small to medium XT. These findings support the use of BTX-A injection for moderate XT, particularly in situations where surgical intervention may be delayed or when concerns regarding anesthesia are present. A recent study (Jiang et al., 2023) reported the outcome of pediatric horizontal concomitant strabismus, including XT, with an overall motor success rate of approximately 60.6%. The study also noted better sensory outcomes in XT compared to esotropia (ET), reinforcing that BTX-A injection can achieve satisfactory alignment and meaningful stereoacuity in children. The present review includes a recent study (Tugcu et al., 2025) on consecutive XT following prior ET treatment, reporting success rate of approximately 73.5% after LR BTX-A injection. The study also noted low rates of transient adverse events such as ptosis and diplopia, supporting BTX-A injection as a safe and effective retreatment option. Across the studies included in this systematic review (Jiang et al., 2023; Spencer et al., 1997; Li & Wu, 2008; Tugcu et al., 2025; Su et al., 2022a; Kunduracıet al., 2022), BTX-A injection adverse effect is mild and transient, such as ptosis, diplopia, vertical deviation, and subconjunctival hemorrhage. Su et al. (2022b) reported that complications were less severe in the BTX-A group compared to the surgical group; they concluded that BTX-A is as effective as surgery for treating IXT in children, although fusion recovery remains lower than with surgery.

#### Occlusion and office-based vergence and anti-suppression therapy (OBVAT) in the management of childhood XT

Short-term evidence indicates that part-time occlusion (2–4 h/day) and OBVAT therapy with home reinforcement can improve control of XT and improve stereoacuity in children with IXT. Across studies (Abdelfatah et al., 2018; Akbari, Mehrpour & Mirmohammadsadeghi, 2021; Pediatric Eye Disease Investigator Group et al., 2015; Pediatric Eye Disease Investigator Group et al., 2014; Ma et al., 2024; Hatt et al., 2023; Ma et al., 2019), mean follow-up was about 4 months, with an overall effective rate of approximately 87%. However, variability in outcome measures such as alignment thresholds (<10 PD), control scoring systems, and stereoacuity limits direct comparison and pooled interpretation.

#### Part-time occlusion

RCT studies by the PEDIG (Pediatric Eye Disease Investigator Group et al., 2015; Pediatric Eye Disease Investigator Group et al., 2014) provide key evidence supporting part-time patching for 3 h daily over a period of 5 months as an effective short-term intervention for improving control in children with XT. More recent RCT evidence from Akbari, Mehrpour & Mirmohammadsadeghi (2021) indicate that alternate patching for 2 hours daily dominant eye 5 days and non-dominant eye 2 days per week can effectively improve control in childhood IXT, supporting the role of personalized occlusion schedules in non-surgical management. Furthermore, a recent meta-analysis of four RCT included 617 subjects confirmed that part-time occlusion provides superior control at both distance and near, reduces distance exodeviation, and improves near stereoacuity compared with observation (Song et al., 2023b). These findings are consistent with Akbari, Mehrpour & Mirmohammadsadeghi (2021) and support part-time occlusion as a practical strategy for improving control in childhood IXT. A pilot RCT reported meaningful improvements in control and stereoacuity with 4 h/day patching over four months, although compliance was a critical determinant of success. Notably, poor adherence significantly reduced the effectiveness of part-time occlusion compared with overminus lens therapy (Abdelfatah et al., 2018). Hatt and colleagues (Hatt et al., 2023) have conducted that part-time patching for 3 h daily may enhance distance control in children with IXT. Furthermore, Han, Han & Han (2023) found that part-time patching was effective in maintaining surgical outcomes and delaying the need for reoperation after BLRc. The benefit was most pronounced in patients with a recurrence of ≤10 PD.

#### Office based vergence and anti-suppression therapy

The role of vision therapy in managing childhood XT has been explored in studies (Ma et al., 2024; Ma et al., 2019), as summarized in Tables 5 and 6. OBVAT therapy demonstrated encouraging outcomes in improving distance control of IXT over a follow-up period of approximately four months (Ma et al., 2024). Similarly, a 12-week program combining OBVAT therapy with home reinforcement yielded significant improvements in distance control and a reduction near IXT (Ma et al., 2019). These findings suggest that structured vision therapy protocols, particularly those incorporating both in-office and home-based components, may offer a valuable non-surgical approach for enhancing control in childhood XT. Further analysis from the same RCT reported significant improvements in fusional vergence ranges, vergence facility, and fusion maintenance, supporting the physiological basis for enhanced control (Ma et al., 2025). In general, both part-time occlusion and OBVAT improve control in the short term compared to observation, but their mechanisms differ. Occlusion primarily acts as anti-suppression therapy and may temporarily reduce the frequency of exodeviation, whereas vision therapy aims to strengthen fusional reserves, vergence facility, and sensory fusion, potentially offering broader benefits for binocular function. Thus, for children with IXT, both part-time occlusion and OBVAT can improve control. Patching may provide a small short-term advantage, while vision therapy offers the potential to enhance fusional function. However, uncertainties remain regarding long-term durability, optimal treatment duration, and patient selection. Future RCTs are needed to establish standardized care pathways for occlusion therapy and vision therapy in the treatment of childhood XT.

### Study limitations

The present systematic review has certain limitations. First, some included studies were retrospective, introducing potential bias. Moreover, the mix of high-quality RCT studies and lower-level observational studies limits clear differentiation of evidence quality and may weaken the interpretability of the conclusions. Second, the definition of treatment success used in this review may introduce bias toward certain management approaches. Because success was primarily defined by a reduction in the angle of deviation, this criterion is more likely for surgical interventions, which directly target ocular alignment. In contrast, non-surgical treatments such as OBVAT and occlusion therapy mainly aim to improve control rather than reduce the deviation angle, making them less likely to meet this success threshold. This methodological limitation should be considered when interpreting comparisons across treatment modalities. Third, the included studies varied in sample size, with some being exceedingly small and others large, which may have increased the risk of random error and affected the precision of pooled estimates. Finally, the heterogeneity in the definitions of treatment success across the included studies. Some studies used a <10 PD benchmark, whereas others used ≤10 PD. Although the present review standardized the threshold to ≤10 PD for consistency, these discrepancies may introduce bias and reduce cross-study comparability. Another limitation of this review is the inability to directly compare the effectiveness of the treatment modalities due to the substantial differences in follow-up durations across interventions. Future research should prioritize conducting RCTs with larger sample sizes, standardized inclusion and exclusion criteria, and consistent treatment protocols. Incorporating long-term follow-up should be essential to assess the durability and overall effectiveness of interventions for childhood XT. Despite these limitations, the present systematic review offers valuable and up-to-date insights into the efficacy of various treatment modalities for improving ocular alignment control in this population.

## Conclusion

Substantial evidence indicates that multiple surgical and non-surgical options are available for managing childhood XT, each offering distinct benefits and limitations. Overminus lenses can improve distance control during testing, but deviation typically return to original state with non-overminus lenses, and their use requires monitoring for possible myopic progression. Extraocular muscle surgery continues to be the primary treatment for constant XT mainly primary infantile XT, with both BLRc and R&R showing similar short-term outcomes, whereas long-term evidence suggests potential advantages for R&R. BTX-A injection offers a minimally invasive alternative for selected cases, achieving satisfactory alignment but with less predictable sensory recovery compared to surgery. Short-term evidence supports part-time occlusion and OBVAT therapy as practical strategies for improving control and binocular function, though uncertainties persist regarding durability, treatment duration, and patient selection. Future RCTs with standardized protocols and long-term follow-up are essential to establish evidence-based care pathways for childhood XT. While the present review provides an overview of the available surgical and non-surgical treatments options for childhood XT, direct comparisons between interventions should be interpreted with caution. The included studies differ significantly in design, sample characteristics, outcome measures, and follow-up durations. Therefore, the review does not support definitive conclusions regarding the comparative effectiveness of one treatment over another. Instead, the findings summarize the evidence within each treatment category without implying superiority across modalities.

##  Supplemental Information

10.7717/peerj.21416/supp-1 Supplemental Information 1 PRISMA checklist

10.7717/peerj.21416/supp-2 Supplemental Information 2 Raw Data for the systematic review

## Additional Information and Declarations

### Data Availability

The following information was supplied regarding data availability: This is a Systematic review/Meta-analysis.
